# Comparative Physicochemical and Functional Properties of Monofloral Honeys from South Korea

**DOI:** 10.3390/foods15060990

**Published:** 2026-03-11

**Authors:** Hyeonjeong Jang, Sukjun Sun, Sungmin Jeong, Sangryul Nam, Sampat Ghosh, Chuleui Jung

**Affiliations:** 1 Department of Plant Medicals, Gyeongkuk National University, Andong 36729, Republic of Korea; jhj971008@naver.com (H.J.); scv6309@naver.com (S.S.); 2 Bee Happy Apiculture Coop., Gunwi 43106, Republic of Korea; t1642@naver.com (S.J.); ulich1355@gmail.com (S.N.); 3 Department of Life Sciences, Sardar Patel University, Balaghat 481331, Madhya Pradesh, India; sampatghosh.bee@gmail.com; 4 Agriculture Research Institute, Gyeongkuk National University, Andong 36729, Republic of Korea

**Keywords:** honey bees, antioxidant capacity, glycemic index, amino acids, *Castanea*, *Robinia*, *Toxicodendron*, *Hovenia*, *Styrax*

## Abstract

Monofloral honeys are widely recognized for their distinct chemical characteristics which are largely influenced by botanical origin. This study aimed to compare the physicochemical and functional properties of monofloral honeys produced in South Korea. Five monofloral honey types, *Castanea crenata*, *Robinia pseudoacacia*, *Toxicodendron* spp., *Hovenia dulcis*, and *Styrax japonicus*, were analyzed, and their floral origins were confirmed through melissopalynological analysis. Physicochemical parameters (moisture content, total soluble solids, hydroxymethylfurfural content, stable carbon isotope ratio, free acidity, pH, color, and sugar composition), along with amino acid profiles, predicted glycemic index (*GI*), and antioxidant activity, were determined. Most physicochemical parameters showed statistically significant differences among honey types. Amino acid composition differed markedly among honey types, with *Castanea* honey exhibiting higher levels of proline, phenylalanine, and leucine compared to *Robinia* and *Styrax* honeys. Predicted *GI* values were predominantly within the low-*GI* range, with no statistically significant differences observed among floral origins. Antioxidant activity showed a similar trend to amino acid content, with *Castanea* honey displaying the highest antioxidant values. These findings demonstrate that botanical origin is a key determinant of the physicochemical and in vitro functional attributes of honey, including antioxidant activity and predicted *GI*, and provide a scientific basis for the characterization of South Korean monofloral honeys.

## 1. Introduction

Honey, mainly blossom honey, is a natural sweet substance produced by honey bees from floral nectar [1]. It is a complex matrix consisting of nearly 200 chemical components, including sugars, water, proteins, organic acids, vitamins, minerals, and phytochemicals [2,3]. Honey quality and chemical composition are influenced by multiple factors, such as bee species, seasonal variation, environmental conditions, beekeeping practices, postharvest storage, and, most importantly, botanical and geographical origins [2,4,5,6].

Despite the recognized importance of the botanical source, previous studies on South Korean monofloral honeys have primarily focused on individual physicochemical parameters, including moisture, sugar composition, hydroxymethylfurfural (HMF), and stable carbon isotope ratio [7,8,9,10]. Comprehensive studies integrating these compositional characteristics with additional parameters such as amino acid profiles, predicted glycemic index (*GI*), and antioxidant activity remain scarce. Available data on less-studied floral types such as *Toxicodendron*, *Hovenia*, and *Styrax* are also limited. During nectar foraging, pollen grains attach to the bodies of honey bees and are transferred into open honeycombs, where they remain in the ripened honey [11,12,13,14]. Melissopalynological analysis, based on the microscopic identification of pollen grains in honey, enables the determination of floral origin [11,15]. This approach allows honey to be classified as monofloral or multifloral and helps identify potential mislabeling of floral sources [16,17]. Although requiring specialized expertise for accurate interpretation, melissopalynological analysis remains a widely accepted and practical tool for verifying the botanical origin of honey [18,19].

The physicochemical characteristics of honey collectively determine its quality, stability, and authenticity. Sugars, as the principal constituents of honey [20], contribute to its caloric value, viscosity, hygroscopicity, and granulation [21]. Moisture content and total soluble solids are closely associated with storage stability and crystallization behavior [22,23,24]. HMF content is commonly used as an indicator of thermal exposure and storage conditions [25], whereas stable carbon isotope ratios (δ^13^C) provide a reliable means of detecting adulteration with C4 plant-derived sugars [26]. Free acidity reflects the degree of honey maturation [6], while pH influences microbial stability and preservation [27,28]. Honey color reflects compositional characteristics and botanical origin [6,29].

Amino acids are important nutritional components of honey, accounting for approximately 1% of its composition [30]. They provide valuable information regarding the botanical origin and quality of honey and are also widely used as indicators of honey adulteration [31,32,33]. Proline, the most abundant amino acid in honey, is predominantly formed during the transformation of floral nectar into honey by honey bees and is widely used as an indicator of honey ripeness [34,35].

*GI* measures the postprandial blood glucose response to carbohydrate intake [36]. Compared with refined sugar, honey generally exhibits a lower glycemic index and energy content, which has been attributed to its compositional complexity [37]. Differences in honey *GI* are largely influenced by sugar composition, particularly the fructose-to-glucose (F/G) ratio, sucrose content, and the sugar-to-oligosaccharide ratio [38]. A previous study has reported a mean *GI* value of around 61 for honey [39]. While *GI* can be determined experimentally in vivo [40,41], it may also be predicted using models based on sugar composition [42]. In the present study, a predictive approach was applied to estimate *GI* values.

Honey contains flavonoids and phenolic compounds that are associated with antioxidant activity and various bioactive effects [6,43,44]. Most antioxidants present in foods originate from plant secondary metabolites, and their antioxidant activity is determined by the chemical structures of the constituent compounds [45,46]. The botanical origin of honey is considered the primary determinant of its antioxidant content, although factors including harvest season, humidity, and bee species may also influence it [47,48,49].

This study aimed to provide comparative and integrated characterization of the physicochemical properties, selected nutritional properties, and functionalities of five monofloral honeys produced in South Korea, with their botanical origins confirmed by melissopalynological analysis, through the evaluation of physicochemical parameters, amino acid profiles, predicted *GI* based on sugar composition, and antioxidant activity.

## 2. Materials and Methods

### 2.1. Honey Samples

A total of 18 monofloral honey samples produced by *Apis mellifera* during the 2025 production season were collected directly from local beekeepers in South Korea, comprising five different floral types. Based on melissopalynological analysis, the monofloral honeys were identified as originating from *Castanea crenata* (*n* = 4), *Robinia pseudoacacia* (*n* = 4), *Toxicodendron* spp. (*n* = 4), *Hovenia dulcis* (*n* = 3), and *Styrax japonicus* (*n* = 3). Detailed information on the samples is provided in Table 1.

### 2.2. Pollen Analysis of Honey

The botanical origin of each honey sample was confirmed prior to further analyses by identifying dominant pollen types following the method described by von der Ohe et al. [50]. Briefly, honey (15 g) was diluted with distilled water and subjected to repeated centrifugation to obtain pollen sediment. The recovered pollen was stained with Safranin-O, washed with ethanol, and suspended in glycerol for microscopic observation. Pollen grains were morphologically identified using a light microscope (BX53, Olympus, Tokyo, Japan), with identification based on published references and validated palynological databases, including PalDat (https://www.paldat.org/, accessed on 24 October 2025) and The Global Pollen Project (https://globalpollenproject.org/, accessed on 24 October 2025).

### 2.3. Physicochemical Analysis

#### 2.3.1. Sugar Content

The concentrations of fructose, glucose, sucrose, and sucrose in honey samples were determined using a modified method described by Jalaludin and Kim [51]. Briefly, honey samples (1 g) were diluted with 50 mL of distilled water and vortex-mixed until completely dissolved. The resulting solutions were filtered through a 0.45 μm nylon syringe filter, and 10 μL of each filtrate was injected into a high-performance liquid chromatography (HPLC) system equipped with a refractive index detector (Agilent 1260, Agilent Technologies, Santa Clara, CA, USA). Chromatographic separation was achieved using an amino column (Asahipak NH2P-50 4E, 250 × 4.6 mm, 5 μm; Shodex, Tokyo, Japan) maintained at 40 °C. The mobile phase consisted of acetonitrile and water (75:25, *v*/*v*), delivered at a flow rate of 1.0 mL min^−1^ with an isocratic elution for 20 min.

#### 2.3.2. Moisture Content and TSS

Moisture content and total soluble solids (TSS) were determined using a digital honey refractometer (ORL 94BS, Kern & Sohn GmbH, Balingen, Germany) at 20 °C. Moisture content was calculated from the refractive index using the Chataway table [52,53], and TSS are expressed as °Brix.

#### 2.3.3. HMF Content

HMF content in honey samples was determined using a modified method based on Khalil et al. [54]. Briefly, honey samples (1 g) were dissolved in 10 mL of distilled water and vortex-mixed until completely homogenized. The resulting solutions were filtered through a 0.45 μm nylon syringe filter, and 20 μL of the filtrate was injected into an HPLC system equipped with a diode array detector set at 280 nm (Agilent 1200, Agilent Technologies, Santa Clara, CA, USA). HMF was separated on a reverse-phase C18 column (CAPCELL PACK C18 MG, 250 × 4.6 mm, 5 μm; Shiseido, Tokyo, Japan) operated at 40 °C. The mobile phase consisted of water and methanol (9:1, *v*/*v*), delivered at a flow rate of 1.0 mL min^−1^ with an isocratic elution for 10 min.

#### 2.3.4. Stable Carbon Isotope Ratio

Stable carbon isotope ratio analysis was performed to determine the ^13^C/^12^C values of honey samples. Approximately 1 mg of each honey sample was weighed into a tin capsule and analyzed using an elemental analyzer (Flash 2000, Thermo Scientific, Bremen, Germany) coupled to an isotope ratio mass spectrometer (DELTA V Advantage, Thermo Scientific, Bremen, Germany). Samples were combusted in the elemental analyzer, and the resulting gases were introduced into the isotope ratio mass spectrometer for isotopic measurement. The analytical procedure was carried out according to previously reported methods [55,56].

#### 2.3.5. Free Acidity and pH

Free acidity was measured by titration to pH 8.30 with 0.1 N NaOH after dissolving 10 g of honey in 75 mL of distilled water, using a calibrated pH meter (Orion Star A211, Thermo Fisher Scientific, Waltham, MA, USA). The pH was determined in a 10% (*w*/*v*) aqueous honey solution using the same pH meter.

#### 2.3.6. Color Determination

Honey color was determined using the Pfund scale [25], with a calibrated portable honey color photometer (HI96785, Hanna Instruments, Seoul, Republic of Korea). The measured values were classified according to the USDA honey color standards [57] as follows: 0–8 mm (water white), 8–17 mm (extra white), 17–34 mm (white), 34–50 mm (extra light amber), 50–85 mm (light amber), 85–114 mm (amber), and >114–140 mm (dark amber).

#### 2.3.7. Amino Acid Profiles

Amino acid analysis of honey samples was performed following the methods of Ghosh et al. [58] and Czernicka et al. [59]. Approximately 0.3 g of sample was hydrolyzed with 15 mL of 6 N HCl under a nitrogen atmosphere at 110 °C for 24 h. After hydrolysis, the samples were concentrated and reconstituted with a citrate buffer (0.12 N, pH 2.20). Amino acid profiles were determined using an amino acid analyzer (S633, Sykam GmbH, Eresing, Germany) equipped with an LCA K07/Li column (4.6 × 150 mm), with photometric detection at 570 and 440 nm.

### 2.4. Functional Properties Analysis

#### 2.4.1. Antioxidant Activity

Total phenolic content (TPC) was determined using the Folin–Ciocalteu method as described by Bertoncelj et al. [60]. Honey samples (5 g) were diluted to 50 mL with distilled water, and 100 μL of the resulting solution was mixed with 1 mL of 10% (*v*/*v*) Folin–Ciocalteu reagent. The mixture was vortexed for 2 min, and absorbance was measured at 750 nm after 20 min against a sugar analogue blank. Gallic acid was used as the reference standard for calibration.

Total antioxidant capacity (TAC) was evaluated using a commercial assay kit (OxiTec™ Total Antioxidant Capacity Assay Kit, Biomax Inc., Guri, Republic of Korea) according to the manufacturer’s instructions. This assay is based on the reduction of Cu^2+^ to Cu^+^ by antioxidant compounds, with Trolox used as the reference standard for calibration.

#### 2.4.2. Predicted *GI*

The *GI* of honey samples was predicted from their sugar composition using the model proposed by Rytz et al. [42], while retaining the original FAO-model structure [61]. The predicted index was calculated as a weighted average based on the relative proportions of major sugars, with values of 20, 100, and 62 assigned to fructose, glucose, and sucrose, respectively. The predicted *GI* was calculated using the equation:

$$GI\;=\;\frac{\sum_{i=1}^{N}x_{i}{GI}_{i}}{\sum_{i=1}^{N}x_{i}}$$

where *x_i_* denotes the relative proportion (%) of each glycemic sugar and *GI_i_* corresponds to its published *GI*. This predictive approach has been demonstrated to yield reliable estimates for carbohydrate-rich foods, including honey.

### 2.5. Statistical Analysis

All analyses were performed in triplicate for each sample, and the results are expressed as mean ± standard deviation (SD). Statistical analyses were conducted using the mean values. All statistical and chemometric analyses were performed using R software (version 4.4.1; R Core Team, Vienna, Austria). Prior to analysis, data normality and homogeneity of variances were evaluated using the Shapiro–Wilk and Levene’s tests, respectively, and all assumptions were satisfied. Differences among honey samples of different floral origins were assessed by one-way analysis of variance (ANOVA), followed by Tukey’s honestly significant difference (HSD) post hoc test. Principal component analysis (PCA) was conducted to explore multivariate relationships among honey samples based on compositional and functional parameters. Statistical significance was set at *p* < 0.05.

## 3. Results

### 3.1. Melissopalynological Analysis

**Figure 1:**
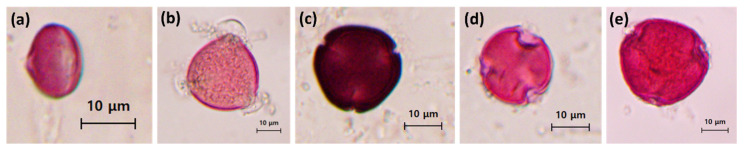
Representative light micrographs (400× magnification) of dominant pollen grains in monofloral honey samples collected in South Korea in 2025: (**a**) *Castanea*, (**b**) *Robinia*, (**c**) *Toxicodendron*, (**d**) *Hovenia*, and (**e**) *Styrax* (scale bars = 10 μm).

Representative light micrographs of the dominant pollen grains identified in each monofloral honey sample are presented in Figure 1. The morphological features were consistent with the respective floral sources, namely *Castanea*, *Robinia*, *Toxicodendron*, *Hovenia*, and *Styrax*. Subsequent quantitative analysis revealed that the dominant pollen types accounted for at least 84% of the total pollen content in all samples (Table 2).

### 3.2. Physicochemical Characteristics

Table 3 indicates that overall sugar composition varied significantly among floral origins (*p* < 0.05). However, post hoc analysis revealed no significant differences in fructose or invert sugar levels. Sucrose content and the F/G ratio also did not differ significantly among honey types. Glucose content was highest in *Styrax* honey, whereas *Castanea* honey exhibited the lowest glucose levels.

The physicochemical properties of the monofloral honey samples are summarized in Table 4. Moisture content, TSS, and HMF levels did not differ significantly among floral origins (*p* > 0.05). The stable carbon isotope ratio showed significant variation among honey types (*p* < 0.05). *Styrax* honey exhibited the lowest δ^13^C (^13^C/^12^C) value, whereas *Robinia* and *Toxicodendron* honeys showed comparatively higher values. Free acidity and pH also differed significantly among floral origins (*p* < 0.01). *Castanea* honey presented the highest free acidity, while *Robinia* and *Styrax* honeys showed the lowest levels. Regarding pH, *Castanea* and *Hovenia* honeys exhibited relatively higher values compared to *Robinia* honey. Color intensity varied markedly among honey types (*p* < 0.001). *Castanea* and *Hovenia* honeys were classified as amber, whereas *Robinia* and *Styrax* honeys were categorized as water white and white, respectively.

The amino acid profiles of the monofloral honey samples are presented in Table 5. Significant differences were observed among floral origins for most amino acids (*p* < 0.05). Total amino acid content varied markedly among honey types (*p* < 0.001), with *Castanea*, *Toxicodendron* and *Hovenia* honeys showing the highest concentration. Proline, the predominant amino acid in all samples, differed significantly among floral origins (*p* < 0.001). *Castanea* and *Hovenia* honeys contained the highest proline level, while *Robinia* honey showed comparatively lower levels. Similar trends were observed for several essential and non-essential amino acids, which were generally higher in *Castanea* and *Toxicodendron* honeys.

### 3.3. Functional Properties

No statistically significant differences were observed in predicted *GI* values among monofloral honeys of different floral origins (*p* > 0.05). *Robinia* honey exhibited the numerically lowest predicted *GI* (51.5 ± 0.5), followed by *Castanea* (51.9 ± 4.0), *Styrax* (53.4 ± 1.3), and *Toxicodendron* (54.7 ± 1.2), whereas *Hovenia* honey showed the highest value (55.6 ± 1.4).

**Figure 2:**
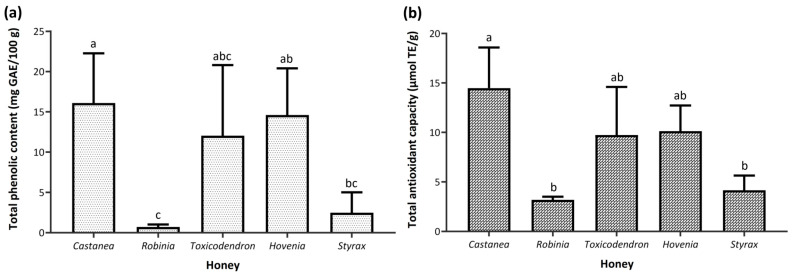
(**a**) Total phenolic content (mg GAE/100 g) and (**b**) total antioxidant capacity (μmol TE/g) of monofloral honey samples collected in Republic of Korea in 2025. Values are presented as mean ± standard deviation (*n* = 3–4 per floral group). Different letters indicate significant differences among floral origins (one-way ANOVA followed by Tukey’s HSD test, *p* < 0.05).

TPC and TAC differed significantly among floral origins (*p* < 0.01) (Figure 2). *Castanea* honey exhibited the highest TPC (16.1 ± 6.2 mg GAE/100 g), whereas *Robinia* honey showed the lowest level (0.7 ± 0.3 mg GAE/100 g). A comparable pattern was observed for TAC, with *Castanea* honey presenting the highest value (14.5 ± 4.1 μmol TE/g), while *Robinia* and Styrax honeys displayed significantly lower values of 3.2 ± 0.3 and 4.2 ± 1.5 μmol TE/g, respectively.

### 3.4. PCA

**Figure 3:**
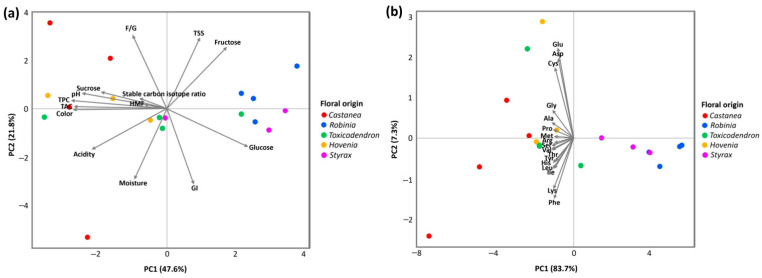
Principal component analysis (PCA) showing the multivariate distribution of monofloral honey samples collected in South Korea in 2025, based on (**a**) all measured parameters excluding amino acids and (**b**) amino acid composition only.

PCA was conducted to explore multivariate patterns among monofloral honey samples based on (a) all measured parameters excluding amino acids and (b) amino acid composition only (Figure 3). In the PCA model constructed using all measured parameters except amino acids (Figure 3a), PC1 explained 47.6% of the total variance, while PC2 accounted for 21.8%, resulting in a cumulative variance of 69.4%. PC1 showed negative correlations with color intensity, free acidity, TPC, and TAC, whereas fructose and glucose contents, TSS, and predicted *GI* were positively associated with this component. PC2 was primarily related to the F/G ratio, TSS, and fructose content. The distribution pattern indicated partial separation among floral origins. *Castanea* and *Hovenia* honeys were positioned along the negative axis of PC1, whereas *Robinia* honey was located in the opposite direction. *Toxicodendron* and *Styrax* honeys occupied intermediate positions, showing some degree of overlap with other groups.

When PCA was performed using amino acid composition only (Figure 3b), the first two principal components explained 83.7% and 7.3% of the total variance, respectively, with a cumulative variance of 91.0%. The separation among samples was largely driven by PC1. *Castanea*, *Toxicodendron*, and *Hovenia* honeys formed a distinct cluster, while *Robinia* and *Styrax* honeys were grouped separately.

## 4. Discussion

### 4.1. Melissopalynological Analysis

Melissopalynological analysis provided clear visual and quantitative evidence for the botanical identification of the analyzed monofloral honey samples [16]. All samples exhibited a dominant pollen frequency exceeding 80%. Generally, honeys containing more than 45% of a single species of pollen are classified as monofloral honey in melissopalynology [62]. Even though *Robinia pseudoacacia* honey is classified as monofloral when the frequency of its pollen exceeding 15–25% [63,64,65], *Castanea* honey requires a much higher threshold, with *Castanea* pollen exceeding 86% for monofloral classification [66,67]. Taken together, these results support a robust classification of the analyzed honeys as monofloral.

### 4.2. Physicochemical Characteristics

Across all honey samples, fructose, glucose, sucrose, and invert sugar contents ranged from 33.8–46.5%, 21.6–33.5%, 0.8–5.8%, and 58.2–77.2%, respectively, which is consistent with the values reported in previous studies on Korean honeys [7,8]. In addition, all samples exhibited a higher proportion of fructose than glucose. Invert sugar and sucrose contents in most samples conformed to the thresholds of ≥60% and ≤5%, respectively, indicating overall compliance with European honey quality standards [68]. In addition, all samples exhibited a higher proportion of fructose than glucose. This observation reflects the general pattern in honey, although exceptions have been reported for specific floral types such as rape and dandelion [6,22]. The fructose-to-glucose (F/G) ratio, which is closely associated with crystallization behavior [12] and glycemic response [38], ranged from 1.2 to 1.9 among the analyzed samples. Although differences in the ratios were not statistically significant, *Castanea* and *Robinia* honeys exhibited numerically higher values. The F/G ratio allows honey samples to be grouped into slow-, medium-, and rapid-crystallizing categories according to thresholds of >1.33, 1.11–1.33, and <1.11, respectively [69]. Accordingly, the F/G ratios observed in this study indicate a tendency toward slow to medium crystallization behavior.

The physicochemical properties of the analyzed monofloral honey samples varied depending on floral origin. Moisture content and total TSS ranged from 15.0 to 23.4% and from 75.1 to 83.1 °Brix, respectively, with no significant differences observed among the samples. Comparable ranges of moisture and TSS have been reported previously, with Indian honeys showing moisture contents of approximately 18–24.5% [70] and Kosovo honeys exhibiting TSS values of 78.60–83.50 °Brix [71]. An inverse relationship between these two parameters has been well documented [23,24]. Moisture content met the EU threshold of 20% in all samples except one [68]. HMF contents were low across all samples and did not differ significantly among floral origins, suggesting minimal thermal degradation and good freshness of the analyzed honeys [6]. All samples remained well below the EU maximum limit of 40 mg/kg for HMF in general honey [68]. The stable carbon isotope ratio showed significant differences among floral origins, with the highest values observed in *Robinia*, *Toxicodendron*, and *Hovenia* honeys, and the lowest values in *Styrax* honey. It enables the detection of sugar adulteration in honey by distinguishing plant sources based on their δ^13^C ratios, with characteristic ranges for C_3_ and C_4_ plants [72,73]. Significant differences in free acidity and pH were observed among the monofloral honeys, with values ranging from 7.0 to 46.9 meq/kg and from 3.3 to 4.6, respectively. These ranges are comparable to those reported previously for Romanian raw honeys by Pașca et al. [74]. None of the samples exceeded the standard limit of 50 meq/kg for free acidity, as defined in EU regulations [68]. Honey color, expressed on the Pfund scale, varied markedly among floral origins, with *Castanea* and *Hovenia* honeys showing the highest color intensity, whereas *Robinia* honey exhibited the lowest values. Honey color is considered an important quality attribute influencing consumer acceptance and preference [6].

*Castanea* honey exhibited the highest amino acid contents, followed by *Toxicodendron* and *Hovenia* honeys, whereas *Robinia* and *Styrax* honeys showed the lowest contents. These findings are consistent with a previous study reporting that amino acid contents and compositions vary according to botanical origin and may serve as potential indicators for the identification of honey origin [33]. In addition, proline was the predominant amino acid in all analyzed honey samples, which is consistent with a previous result documented by Iglesias et al. [75]. Proline is largely derived from the salivary secretions of honey bees during honey formation from nectar [6,75]. It is also known to play an important role in insect flight metabolism and, in addition to carbohydrates, to serve as a metabolic fuel during flight [76,77]. However, the absolute concentrations of individual amino acids in honey are relatively low, and their nutritional relevance at typical consumption levels remains limited.

### 4.3. Functional Properties

Foods are generally classified as high-*GI* (≥70), medium-*GI* (56–69), or low-*GI* (≤55) [78]. Based on this classification, all analyzed honey samples were categorized as low-*GI* foods, with the exception of *Hovenia* honey, which fell within the medium-*GI* range. The *GI* of honey has been reported to vary widely depending on botanical origin, ranging from 14.3 to 109.0, with most honey samples classified as low- to medium-*GI* foods [40]. The predicted *GI* values obtained in the present study fall within this reported range. Rytz et al. [42] proposed a compositional model for estimating *GI* values of carbohydrate-rich foods based on sugar profiles. While such predictive models provide a practical approach for estimating glycemic potential from compositional data, direct validation studies comparing predicted and experimentally determined *GI* values specifically in honey remain limited. Therefore, the predicted *GI* values presented in this study should be interpreted as compositional estimations rather than direct indicators of in vivo glycemic response.

The antioxidant activities of the analyzed monofloral honey samples, evaluated based on TPC and TAC, differed significantly according to floral origin. TPC values showed clear variation among honey types, with *Castanea* honey exhibiting the highest content, whereas *Robinia* honey displayed the lowest values. Similar TPC values were reported by Kang et al. [79], ranging from 2.35 to 19.96 mg GAE/100 g. A similar trend was observed for TAC, with *Castanea* honey showing the highest value, while *Robinia* and *Styrax* honeys exhibited significantly lower values. The observed values are consistent with those reported in earlier studies on honeys of different botanical origins, with reported ranges of 13.60–30.72 µmol TE/g and 6.42–9.43 nmol TE/mg, respectively [80,81]. The antioxidant content of honey is strongly influenced by the plant species selected by honey bees [70]. The Folin–Ciocalteu assay measures total reducing capacity and is not specific to phenolic compounds, as the reagent also reacts with various non-phenolic reducing substances. Nevertheless, it provides a reasonable approximation of total phenolic content in most plant-derived samples, where phenolics are typically the predominant antioxidants [82]. A strong correlation between total phenolic content and antioxidant activity was reported by Beretta et al. [83], while phenolic content has also been shown to correlate positively with honey color intensity [84]. Saxena et al. [85] demonstrated that both proline and phenolic contents contribute to the antioxidant activity of honey. The botanical origin of honey significantly influences its chemical composition, including phenolic acids, thereby shaping its antioxidant properties [81,86,87].

Based on the present results, the reported functional properties of each monofloral honey are further discussed. Kwon et al. [88] reported that *Castanea* honey exhibits potential antiviral activity and may act as a useful immunomodulator. In addition, significantly higher antioxidant capacities have been reported for chestnut honey [89], which is consistent with the findings of the present study. *Robinia* honey has been reported to exhibit greater anti-inflammatory potential than the other honeys evaluated [90]. *Hovenia* honey demonstrated significant antioxidant activities, along with antibacterial activity against both Gram-positive and Gram-negative bacteria [91]. In contrast, although some indirect evidence may be inferred from botanical sources, direct investigations into the functional properties of *Styrax* and *Toxicodendron* honeys remain very limited.

### 4.4. PCA

In the PCA model excluding amino acids, variation associated with antioxidant-related parameters and color intensity contributed substantially to sample differentiation, resulting in a clear separation of *Castanea* and *Hovenia* honeys from *Robinia* honey. This pattern suggests that darker honeys with greater antioxidant capacity exhibit compositional profiles distinct from those of lighter honeys, consistent with previous findings linking honey color to antioxidant properties [84]. Comparable differentiation based on physicochemical composition has also been reported in an earlier study [92]. In contrast, PCA based on amino acid composition revealed a more distinct clustering pattern, with *Castanea*, *Toxicodendron*, and *Hovenia* honeys clearly separated from *Robinia* and *Styrax* honeys. The primary axis of variation captured differences in overall amino acid abundance, distinguishing samples with higher concentrations from those with lower concentrations. These findings are consistent with previous studies indicating that amino acid profiles may serve as useful indicators for the differentiation of botanical origin in monofloral honeys [33].

Despite these findings, several limitations should be acknowledged. First, the relatively small sample size (*n* = 3–4 per floral group) may limit statistical power and the broader generalizability of the results. Second, as samples were collected within a single year, the findings may not fully capture potential year-to-year variability in honey composition. Environmental factors, including climate conditions, soil characteristics, and regional floral diversity, may also influence physicochemical and compositional parameters [2,4,5,6]. Third, the predicted *GI* values were estimated using a compositional model based on sugar profiles rather than determined through in vivo glycemic response testing, and may not fully reflect actual physiological responses. Future studies incorporating larger, geographically stratified sample sets, multi-year sampling, and experimental validation of *GI* values are warranted to further strengthen and refine these findings.

## 5. Conclusions

This study provides an integrated characterization of five South Korean monofloral honeys by combining melissopalynological confirmation with physicochemical and in vitro functional analyses. Significant differences among floral origins were observed in several physicochemical parameters, including free acidity, pH, color, stable carbon isotope ratio, and sugar composition. Honey type was also associated with variations in amino acid composition and in vitro antioxidant-related parameters. Although predicted *GI* values showed limited variation among floral types, these results represent compositional estimations derived from sugar profiles rather than direct measurement of in vivo glycemic responses. The relatively small sample size and the reliance on predictive and in vitro approaches constrain the broader generalizability of the findings. Future studies incorporating larger, geographically and temporally stratified datasets, along with experimental validation of functional and glycemic properties, are required to clarify the biological relevance of the observed compositional differences.

## Figures and Tables

**Table 1:** Sampling sites, geographic coordinates, and landscape types of monofloral honey samples collected in South Korea in 2025.

Floral Source	City	Latitude (°N)	Longitude (°E)	Landscape Type
*Castanea*	Cheongyang	36.3559	126.7936	Forest–agricultural
	Andong	36.6187	128.8271	Forest–agricultural
	Sangju	36.3799	128.0251	Mountainous forest
	Gyeongju	35.6802	129.4525	Agricultural
*Robinia*	Daegu	36.2550	128.5579	Forest–agricultural
	Gyeongju	35.6802	129.4525	Agricultural
	Sangju	36.3799	128.0251	Mountainous forest
	Andong	36.7503	128.7685	Forest–agricultural
*Toxicodendron*	Sangju	36.3799	128.0251	Mountainous forest
	Gimcheon	36.0435	127.9640	Forest–agricultural
	Sangju	36.5071	128.2084	Agricultural
	Yeongwol	37.2378	128.4178	Forest–agricultural
*Hovenia*	Sangju	36.5071	128.2084	Agricultural
	Yeongwol	37.2378	128.4178	Forest–agricultural
	Andong	36.6139	128.7943	Forest–agricultural
*Styrax*	Miryang	35.5001	128.6273	Agricultural
	Hampyeong	35.1251	126.5848	Forest–agricultural
	Sancheong	35.2914	127.8284	Forest–agricultural

**Table 2:** Floral origin and pollen frequency (%) of monofloral honey samples collected in South Korea in 2025, based on melissopalynological analysis.

Floral Source		N	Pollen Frequency (%)
Family	Genus		
Fagaceae	*Castanea*	4	87.5 ± 7.5
Fabaceae	*Robinia*	4	87.3 ± 12.0
Anacardiaceae	*Toxicodendron*	4	84.5 ± 9.6
Rhamnaceae	*Hovenia*	3	84.3 ± 13.7
Styracaceae	*Styrax*	3	86.4 ± 4.9

Values are presented as mean ± standard deviation.

**Table 3:** Major sugar contents (%) and fructose-to-glucose (F/G) ratios of monofloral honey samples collected in South Korea in 2025.

	*Castanea*	*Robinia*	*Toxicodendron*	*Hovenia*	*Styrax*	*F*_(4,13)_	*p*
Fructose (%)	38.3 ± 4.5 a	44.0 ± 1.8 a	38.3 ± 2.4 a	37.5 ± 2.1 a	39.5 ± 1.7 a	3.347	<0.05
Glucose (%)	24.5 ± 3.3 b	28.2 ± 1.7 ab	28.9 ± 2.9 ab	25.9 ± 2.0 ab	31.3 ± 2.9 a	3.348	<0.05
Sucrose (%)	3.6 ± 1.9	2.1 ± 0.9	3.0 ± 1.3	3.1 ± 2.1	4.5 ± 1.4	1.084	0.404
Invert sugar (%)	62.8 ± 3.8 a	72.2 ± 3.3 a	64.9 ± 6.7 a	63.4 ± 3.4 a	70.9 ± 4.1 a	4.104	<0.05
F/G	1.6 ± 0.3	1.6 ± 0.1	1.4 ± 0.2	1.4 ± 0.1	1.3 ± 0.1	2.369	0.106

Values are presented as mean ± standard deviation (*n* = 3–4 per floral group). Different letters within each row indicate significant differences among floral groups (one-way ANOVA followed by Tukey’s HSD test, *p* < 0.05).

**Table 4:** Physicochemical properties of monofloral honey samples collected in South Korea in 2025.

	*Castanea*	*Robinia*	*Toxicodendron*	*Hovenia*	*Styrax*	*F*_(4,13)_	*p*
Moisture (%)	18.6 ± 3.4	17.1 ± 1.4	17.7 ± 1.0	17.8 ± 0.4	17.1 ± 1.2	0.427	0.787
TSS (°Brix)	79.8 ± 3.2	81.2 ± 1.3	80.6 ± 0.9	80.5 ± 0.4	81.2 ± 1.2	0.431	0.784
HMF (mg/kg)	1.5 ± 2.9	5.1 ± 4.4	0.1 ± 0.1	15.0 ± 13.4	13.9 ± 20.5	1.667	0.217
Stable carbon isotope ratio (‰)	−26.3 ± 0.4 ab	−25.6 ± 0.4 a	−25.3 ± 0.5 a	−25.3 ± 0.7 a	−27.3 ± 1.4 b	4.846	<0.05
Free acidity (meq/kg)	32.2 ± 10.8 a	12.4 ± 3.8 b	25.7 ± 9.0 ab	26.6 ± 5.5 ab	11.3 ± 3.8 b	5.535	<0.01
pH	4.3 ± 0.3 a	3.6 ± 0.2 b	4.1 ± 0.3 ab	4.2 ± 0.1 a	3.9 ± 0.2 ab	5.137	<0.01
Color (Pfund scale; mm)	97.0 ± 24.1 a (Amber)	4.8 ± 3.9 c (Water white)	63.8 ± 20.8 ab (Light amber)	87.0 ± 16.7 a (Amber)	26.7 ± 18.7 bc (White)	17.126	<0.001

Values are presented as mean ± standard deviation (*n* = 3–4 per floral group). Different letters within each row indicate significant differences among floral groups (one-way ANOVA followed by Tukey’s HSD test, *p* < 0.05).

**Table 5:** Amino acid profiles (mg/kg) of monofloral honey samples collected in South Korea in 2025.

	*Castanea*	*Robinia*	*Toxicodendron*	*Hovenia*	*Styrax*	*F*_(4,13)_	*p*
Asp	371.8 ±33.9 ab	154.4 ± 17.7 b	348.8 ± 30.6 ab	463.8 ± 222.9 a	192.8 ± 58.6 b	6.582	<0.01
Thr	100.6 ± 18.3 a	37.0 ± 7.8 c	80.2 ± 8.0 ab	83.1 ± 5.2 a	54.1 ± 9.0 bc	19.870	<0.001
Ser	157.8 ± 21.0 a	56.8 ± 11.8 c	119.5 ± 15.6 b	119.2 ± 8.3 b	81.7 ± 11.2 c	26.602	<0.001
Glu	512.2 ± 200.1 a	117.3 ± 28.4 a	463.8 ± 239.4 a	457.1 ± 172.3 a	169.0 ± 62.2 a	4.550	<0.05
Pro	775.6 ± 146.2 a	272.7 ± 27.7 c	594.5 ± 51.9 ab	604.3 ± 97.2 a	376.2 ± 92.1 bc	17.966	<0.001
Gly	110.9 ± 16.7 a	35.9 ± 9.1 c	100.9 ± 29.8 a	91.7 ± 3.3 ab	51.6 ± 12.2 bc	12.886	<0.001
Ala	119.5 ± 18.3 a	36.4 ± 10.9 b	97.7 ± 14.1 a	102.8 ± 5.0 a	58.9 ± 12.0 b	25.249	<0.001
Val	130.4 ± 23.0 a	47.9 ± 9.1 b	105.2 ± 10.3 a	107.8 ± 7.3 a	67.9 ± 12.0 b	21.521	<0.001
Cys	55.4 ± 2.6 ab	10.1 ± 2.4 c	47.5 ± 17.0 ab	57.2 ± 13.3 a	31.7 ± 4.8 bc	14.546	<0.001
Met	44.8 ± 8.3 a	16.3 ± 4.4 c	30.1 ± 4.3 b	32.1 ± 2.3 ab	22.2 ± 5.0 bc	15.543	<0.001
Ile	101.6 ± 24.9 a	35.7 ± 8.4 c	77.2 ± 11.4 ab	80.1 ± 6.6 ab	52.3 ± 7.6 bc	12.374	<0.001
Leu	163.1 ± 39.6 a	61.2 ± 15.9 c	126.4 ± 16.0 ab	132.8 ± 10.3 ab	86.6 ± 13.8 bc	11.755	<0.001
Tyr	74.9 ± 33.9 a	22.2 ± 4.0 b	45.9 ± 14.3 ab	37.3 ± 2.5 ab	22.6 ± 4.7 b	5.582	<0.01
Phe	157.8 ± 60.0 a	40.2 ± 8.7 b	108.4 ± 16.1 ab	101.3 ± 21.6 ab	59.6 ± 12.3 b	8.072	<0.01
His	18.7 ± 4.0 a	7.4 ± 1.4 c	14.3 ± 1.6 ab	13.9 ± 1.8 ab	9.7 ± 1.7 bc	12.799	<0.001
Lys	142.6 ± 40.9 a	55.6 ± 13.1 c	112.6 ± 9.3 ab	97.4 ± 18.3 abc	75.1 ± 4.3 bc	8.792	<0.01
Arg	97.9 ± 13.3 a	31.9 ± 10.0 d	74.1 ± 15.5 ab	63.4 ± 10.8 bc	37.0 ± 9.3 cd	18.690	<0.001
Total	3135.7 ± 420.0 a	1039.1 ± 142.0 b	2546.9 ± 391.1 a	2645.4 ± 268.3 a	1448.9 ± 328.2 b	26.896	<0.001

Asp, aspartic acid; Thr, threonine; Ser, serine; Glu, glutamic acid; Pro, proline; Gly, glycine; Ala, alanine; Val, valine; Cys, cysteine; Met, methionine; Ile, isoleucine; Leu, leucine; Tyr, tyrosine; Phe, phenylalanine; His, histidine; Lys, lysine; Arg, arginine. Values are presented as mean ± standard deviation (*n* = 3–4 per floral group). Different letters within each row indicate significant differences among floral groups (one-way ANOVA followed by Tukey’s HSD test, *p* < 0.05).

## Data Availability

The original contributions presented in this study are included in the article. Further inquiries can be directed towards the corresponding author.
